# The Influence of Genetic Polymorphisms of *IL33* and *IL1RL1* Genes on the Immunopathogenesis of Periodontitis

**DOI:** 10.1155/ijod/7599713

**Published:** 2025-05-02

**Authors:** Aléia Harumi Uchibaba Yamanaka, Josiane Bazzo de Alencar, Victor Hugo de Souza, Joana Maiara Valentini Zacarias, Larissa Danielle Bahls-Pinto, Cléverson O. Silva, Ana Maria Sell, Quirino Alves de Lima Neto

**Affiliations:** ^1^Department of Clinical Analysis and Biomedicine, Maringá State University, Maringá, Paraná, Brazil; ^2^Department of Basic Chairs, Marília Medical School–FAMEMA, Marília, São Paulo, Brazil; ^3^Department of Dentistry, Maringá State University, Maringá, Paraná, Brazil; ^4^Department of Basic Health Sciences, Maringá State University, Maringá, Paraná, Brazil

## Abstract

Periodontitis (PD) is an inflammatory disease that affects the protective and supporting tissues of teeth. Mutations in cytokines and their receptors may influence the immunopathogenesis of PD, but the role of interleukin-33 (IL-33) and IL1RL1 is not clear. Thus, this study aimed to analyze the polymorphisms in *IL33* (rs1929992 and rs7025417) and *IL1RL1* (rs11685424 and rs3821204) genes and the IL-33 serum levels in PD patients. A case–control study was performed with 186 PD patients and 189 controls. Genotyping was performed by polymerase chain reaction using sequence-specific primer (PCR-SSP) technique. Serum levels of IL-33 were determined using the immunoenzymatic method. Statistical analyses were performed using SNPStats and OpenEpi. *p* > 0.05 was considered statistically significant. The *IL33* rs7025417 C/C genotype was a risk factor for PD in nonsmokers (*p*=0.0015) regardless of smoking status and gender. In the general population, the *IL1RL1* rs3821204 G/G genotype was protective for PD (*p*=0.006), regardless of gender. Nevertheless, the IL-33 serum levels were increased in patients compared to controls (*p* < 0.0001); however, no difference was observed among PD patients. The polymorphisms *IL33* rs7025417 and *IL1RL1* rs3821204 were associated with risk and protection, respectively, and the production of IL-33 was higher in PD patients than in controls, independent of the extent or severity of the disease.

## 1. Introduction

The oral cavity and periodontal tissues are constantly exposed to pathogens that may activate the immunological system [1]. Dysbiosis of the oral microbiota, particularly of the subgingival biofilm, leads to an inflammatory response characterized by the infiltration of immune cells and the release of cytokines involved in activating effectors that promote tissue destruction [2]. Therefore, the disruption of homeostasis by oral microbiota may result in the deterioration of both soft and hard periodontal tissues, including the gums, periodontal ligament, and alveolar bone [1].

Cytokines and their receptors may play a key role in the inflammatory response of periodontitis (PD) [3]. Interleukin-33 (IL-33), a member of the IL-1 cytokine family, is constitutively expressed and stored in the nucleus of endothelial and epithelial cells. However, under cellular damage, IL-33 is released into the extracellular space as an alarmin [4]. The IL-33 receptor has distinct isoforms: a secreted soluble form (sST2) and a membrane-bound form (ST2L). The sST2 receptor acts as a decoy receptor neutralizing IL-33 activity [4], and sST2 may attenuate the symptoms of rheumatoid arthritis by inhibiting the release of proinflammatory cytokines [5]. The interaction of IL-33 and ST2L is involved in Th2 immunity and allergic inflammation [6]; however, it extends beyond Th2 responses. Indeed, it plays a crucial role in the activation of immune cells involved in Th1 responses, chronic inflammation, and infection [4]. Moreover, the IL-33/ST2L axis can increase RANKL expression and decrease OPG, favoring the RANK/RANKL interaction and promoting bone loss [7]. The presence of *Porphyromonas gingivalis* leds to the increased expression of IL-33 through gingipain-dependent mechanisms [8], followed by the bone resorption process, suggesting a synergistic effect during osteoclastogenesis [9].

Single nucleotide polymorphisms (SNPs) are the most frequent mutations within the genome. These mutations result from the exchange of a single nucleotide and can alter the quantity and conformation of proteins. Several SNPs in *IL33* and *IL1RL1* genes were associated with PD [10, 11], as well as polymorphisms in other genes [3]. Thus, the objective of this study was to analyze the influence of polymorphisms in the *IL33* (rs1929992 T >C and rs7025417 T >C) and *IL1RL1* (rs11685424 G >A and rs3821204 C >G) genes, as well as the serum levels of the IL-33 cytokine, in the pathogenesis of PD in individuals residents in the north/northeast region of Paraná, Brazil.

## 2. Materials and Methods

### 2.1. Sample Selection

This case–control study included 375 (case/control: 186/189) individuals from the north and northeast of Paraná, southern Brazil. All were selected by a specialized periodontist at the State University of Maringá (UEM) and Ingá University Center (UNINGÁ) from 2012 to 2021 and signed a free and informed consent form. This study was approved by the Human Research Ethics Committee of the State University of Maringá (UEM-COPEP No. 719/2011, 12/02/2011 and CAAE/2016 No. 61544916.4.000.0104).

Participants were assessed for probing depth (PDe), bleeding on probing (BOP), and clinical attachment loss (CAL) at four sites per tooth (mesial, vestibular, distal, and lingual) and classified according to the International Workshop for Classification of Periodontal Diseases and Conditions (1999). In this classification, PD patients were required to present at least five sites on different teeth with PDe ≥5 mm, CAL ≥3 mm, and more than 25% BOP. The disease was categorized by severity and extent, where extent was defined as localized (≤30% of sites affected) or generalized (>30% of sites affected). Severity was classified as slight (CAL 1–2 mm), moderate (CAL 3–4 mm), or severe (CAL >5 mm) [12]. Although the 1999 classification was used for patient selection and characterization, these individuals would correspond to stages II and III (based on severity, complexity, extension, and distribution) and grade B (moderate rate of progression) according to the 2017 classification of periodontal diseases [13]. The control group consisted solely of periodontally healthy individuals with no gingival inflammation, no PDes >4 mm, and less than 25% BOP.

All individuals selected in this study were over 30 years old and had at least 20 teeth in the oral cavity. Individuals who had diabetes mellitus, acute infections, bone involvement diseases, gingivitis, and aggressive PD, who used antibiotics or anti-inflammatories, and who underwent periodontal treatment in the last 6 months were not included in this study. The ethnic group and smoking history of those selected were determined by self-report. The population in this study was classified as a mixed population, according to the great miscegenation process that occurred in the state of Paraná [14]. Regarding smoking habits, individuals who could not estimate the date, who abstained from smoking, or who stopped less than 10 years ago were included in the group of smokers [15].

### 2.2. DNA Extraction

DNA extraction was performed using the QIAamp DNA blood mini kit (Qiagen, Valencia, CA, USA) in accordance with the manufacturer's instructions. DNA concentrations and quality were assessed by optical density using the ThermoScientific Nanodrop 2000 equipment (Nanodrop Technologies, Wilmington, DE, USA).

### 2.3. Genotype Analysis and Visualization of the Amplified Product

The genotyping was performed using polymerase chain reaction using sequence-specific primers (PCR-SSPs). The primer sequences were designed and tested on Primer Blast (NCBI) (https://blast.ncbi.nlm.nih.gov/blast.cgi). Primer sequences used were as follows: for *IL33* rs1929992 - F: 5′ CATTTTCCCCCCAAATTTCAAT/C 3′, R: 5′ AAGTCATCATCAACTTGGAACC 3′; *IL33* rs7025417 - F: 5′ GTATAACAAAACAGTCTCAGAACT/C 3′, R: 5′ ACTGTAGTCTTTTATCCCTTACC 3′; *IL1RL1* rs3821204 - F: 5′ GCATGGTCCGTTCTATACC/G 3′, R: 5′ AGTAGCACCTAGGATTTTCTCAC 3′; *IL1RL1* rs11685424 - F: 5′CTCCTGAGTAGCTTGGATTAG 3′, R: 5′ CAGTAACCTATGGAGGATGC/T 3′. As an internal reaction control, primers for the human growth hormone (hGH) gene were included, amplifying a 434 bp fragment.

The amplifications were performed in a thermocycler (System 9700 Applied Biosystems, Foster City, CA, USA). The PCR products were visualized by agarose gel electrophoresis with the SYBR Safe DNA Gel Stain (Invitrogen Life Technologies, Grand Island, NY, USA). The bands were analyzed by comparison with a 100 bp molecular size marker (Invitrogen Life Technologies, Grand Island, NY, USA).

The quality control of the reactions was ensured by direct sequencing performed on two samples of each variant, using BigDye terminator v3.1 cycle sequencing kit (Applied Biosystems, Thermo Fisher Scientific, USA) in accordance with the manufacturer's instructions on an automated analyzer (Applied Biosystems 3500xL).

### 2.4. Determination of the IL-33 Serum Levels

IL-33 levels in patient and control serum were assessed using an enzyme-linked immunosorbent assay (ELISA), which relies on antigen–antibody binding, resulting in a color change due to the addition of a substrate, typically TMB (3,3′, 5,5′-tetramethylbenzidine). This technique is widely used for detecting and quantifying soluble antibodies, antigens, proteins, and glycoproteins in biological samples [16]. The quantification of IL-33 was performed using a Human IL-33 ELISA Kit (Thermo Fisher Scientific, Waltham, MA, USA) in accordance with the manufacturer's instructions. The absorbance of each well was read at 450 nm using an ELISA reader (Asys Hitech GmbH, Eugendorf, Austria).

The test included 30 patients and 8 controls, nonsmokers, matched by gender and age. Exclusion criteria were no use of anti-inflammatory drugs, no antibiotics in the last 6 months, and no periodontal treatment during the same period. Patients were classified based on severity: 15 had generalized and 15 had localized PD. Regarding extent, 6 patients were classified as having slight PD, 10 as moderate, and 14 as severe.

### 2.5. Statistical Analysis

All analyses were performed considering the existence of three groups: smokers, nonsmokers, and general population. The QUANTO software version 1.2.4. was used to calculate the statistical power of 80%.

The association between genetic polymorphisms and PD was evaluated using the Chi-square test with Yates correction or Fisher's exact test. The false discovery rate (FDR) correction was applied for statistically significant results. The effect of age, gender, and smoking status was adjusted by multivariate logistic regression. Odds ratio (OR) was analyzed only for statistically significant *p*-values, with 95% confidence intervals (CIs = 95%). For these analyses and calculating Hardy–Weinberg equilibrium, the OpenEpi program Version 2.3.1 and SNPStatus software (http://bioinfo.iconcologia.net/index.php) were used.

The comparison of cytokine serum levels between groups was evaluated using the BioEstat 5.0 program. The Shapiro–Wilk test was used to assess the normality of the samples, and Student's *t*-test was chosen to assess the correlation between groups. For all tests, *p* < 0.05 was considered statistically significant.

## 3. Results

For this study, 375 individuals were analyzed, and 50.4% of them were controls. Men were 43.55% of patients and 39.15% of the control group. The mean age was 46.73 ± 9.09 and 45.17 ± 9.09 years for patients and controls, respectively. There were no differences in the distribution of genders and mean age between patients with PD and controls (*p* > 0.05), suggesting adequate matching between cases and controls. Most of the individuals who participated in the study were nonsmokers (58.1% for patients and 76.7% for controls), and a higher frequency of smokers was observed among individuals with PD than controls, conferring risk for disease development (41.9% vs. 23.3%, OR = 2.38, CI = 1.52–3.71, *p*=0.0001). Eighty-seven patients were classified according to the extent and severity of the disease: about half of them had the generalized or localized form and, regarding severity, 26.5% had slight, 40.2% moderate, and 33.3% severe PD. The characteristics of the studied population are shown in Table 1.

The genotype frequency distributions for all analyzed genes were in accordance with the Hardy–Weinberg equilibrium (*p* > 0.05).

The *IL33* rs1929992 and rs7025417 SNPs were in high linkage disequilibrium (*D*′ = 0.85, *p* < 0.00001) as were *IL1RL1* rs11685424 and rs3821204 SNPs (*D*′ = 0.7433, *p* < 0.0001), which suggests that these alleles are frequently inherited together.

The *IL33* rs7025417 C allele was more frequent in patients (22%) compared to controls (17%), even with no statistical difference (*p*=0.06). There were no significant differences in the *IL33* rs7025417 genotype distribution between all patients and controls (*p* > 0.05).

The analyses of the genotype and allele frequency, considering the smoking habit, revealed that in nonsmokers, the SNP *IL33* rs7025417 C/C was associated with the risk of PD in codominant and recessive models. In the recessive inheritance model, individuals with the *IL33* rs7025417 C/C genotype were more susceptible to developing PD compared to individuals with the *IL33* rs7025417 T/C and T/T genotype (OR = 12.86, CI = 1.60–103.49, *p*=0.001) (Table 2). However, the same was not observed in smokers. Therefore, the data suggest that *IL33* rs7025417 was associated with PD, and smoking habits may be a confounding factor.

The *IL1RL1* rs3821204 SNP was associated with the protection against the development of PD in the codominant and recessive models in general population (Table 2). In the recessive inheritance model, individuals with the *IL1RL1* rs3821204 G/G genotype were less susceptible to developing PD compared to individuals with the *IL1RL1* rs3821204 C/C and C/G genotypes (OR = 0.19, CI = 0.05–0.72, *p*=0.006). After stratification according to smoking habits, the protective effect was lost for nonsmokers and smokers due to a lack of statistical power (>75%). These data suggest that *IL1RL1* rs3821204 may be associated with PD independent of smoking habits.

In the general population, for *IL33* rs1929992, the T/T genotype was the most frequent among both patients and controls, followed by T/C and C/C, respectively. Similarly, for *IL33* rs7025417, the wild-type genotype was the most common, followed by the heterozygous and mutated homozygous genotypes. Regarding *IL1RL1* rs11685424, the G/A genotype was the most frequent, while G/G and A/A had similar frequencies in both patients and controls (Table 2). The *IL33* rs1929992 and *IL1RL1* rs11685424 SNPs showed no association with PD in any of the analyzed groups. The supporting tables present the distribution of genotype frequencies for nonsmokers (Table S4) and smoker groups (Table S5).

Stratification by gender showed no association with the disease for all analyzed SNPs in nonsmokers (Table S1), smokers (Table S2), and the general population (Table S3).

**Figure 1:**
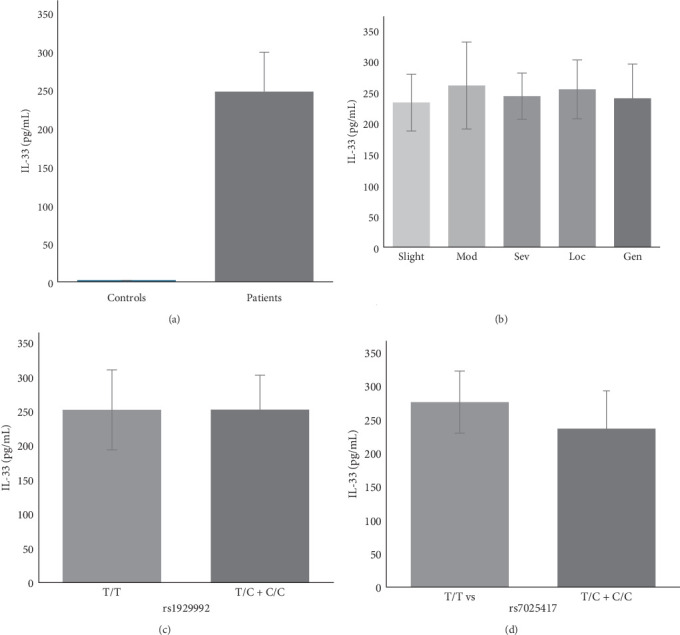
Serum levels of interleukin-33 (IL-33) in nonsmokers patients with periodontitis (PD) and controls. Comparison of IL-33 concentrations: (A) between patients and controls; (B) between groups of patients considering extension and severity; (C) among patients with the T/T vs. T/C + C/C genotype of *IL33* rs1929992; (D) between patients with the T/T vs. T/C + C/C genotype of *IL33* rs7025417. Gen, generalized; Loc, localized; Mod, moderate; Sev, severe.

Serum levels of IL-33 were measured for 30 nonsmoking PD patients and 8 controls, aged between 31 and 63 years old, and matched by age and gender. Patients were classified according to the severity of the disease (slight, moderate, and severe) and extent (localized and generalized). The serum levels of IL-33 were increased in PD patients when compared to controls (mean ± SD: 247.0 ± 51.2 pg/mL vs. 0.0 ± 0.0 pg/mL; *p* < 0.0001) (Figure 1A). Nevertheless, IL-33 levels in controls were lower than the detection limit, as expected in individuals without immune disorders. No differences were found when patients were grouped according to severity and extent within PD (Figure 1B), which may indicate a contribution of other factors in disease progression. No differences were observed comparing genotypes and cytokine production for *IL33* rs1929992 (Figure 1C) and *IL33* rs7025417 polymorphisms (Figure 1D). However, the *IL33* rs7025417 T/T genotype showed increased levels of IL-33 when compared to the T/C + C/C genotypes, even without a statistical difference (*p*=0.06). The serum concentrations of the studied population are presented in Table 3.

## 4. Discussion

This study found a possible association between polymorphisms of *IL33* and *IL1RL1* and PD. The *IL33* rs7025417 C/C genotype showed lower production of IL-33 and was associated with an increased risk of PD development in the recessive inheritance models regardless of smoking and gender. Additionally, the *IL1RL1* rs3821204 G/G genotype was associated with protection against disease in the same inheritance model, regardless of gender.

Smoking habits were a risk factor for PD in this study. PD is a multifactorial disease, and several biological and environmental factors have been associated with the risk of developing PD, such as genetic background and smoking habits [1]. Smokers had decreased T cells and NK cells, which are regulatory cytokines that reflect the immunosuppressive effects, which may contribute to enhanced susceptibility to PD [17].

In our control group, the genotype distribution of *IL33* rs1929992 was 39.3% for T/T, 45.5% for T/C, and 15.2% for C/C, closely resembling the findings of Pan et al. [18], which reported frequencies of 28%, 49.2%, and 22.8%, respectively. Similarly, for *IL33* rs7025417, the genotype frequencies in our control group were 68.8% for T/T, 29.1% for T/C, and 2.1% for C/C, aligning with the results from Prieto-Penha et al. in Spain [19] (70.8% for T/T, 25.9% for T/C, and 3.3% for C/C), but differing substantially from those reported by Wei et al. [20] (28.9% for T/T, 48.5% for T/C, and 22.6% for C/C). Regarding *IL1RL1* rs11685424, the genotype distribution in our control group was 24.8% for A/A, 53.1% for A/G, and 22.1% for G/G, closely matching those reported in [20], which were 28.2%, 49.2%, and 22.6%, respectively. In contrast, the genotype frequencies for *IL1RL1* rs3821204 in our control group (66.9% for C/C, 29% for C/G, and 4.1% for G/G) differed markedly from those observed in the study by Pan et al. [18], which reported 47.1%, 41%, and 11.9%, respectively.

The differences observed in the distribution of genotype frequencies may be explained by the historical admixture process that occurred in Paraná. The population of Paraná is primarily of European descent, with additional genetic contributions from African and Amerindian ancestry. Due to these genetic differences, associations with diseases may vary across populations. Therefore, studying genetic variations in Paraná may provide valuable insights into the genetic basis of PD pathogenesis.

In this study, the *IL33* rs7025417 SNP was associated with an 11-fold risk of developing PD in nonsmokers. The *IL33* rs7025417 C/C genotype and C allele have shown lower production of IL-33 when compared with the T/T genotype or T allele [21]. The decreased levels of IL-33 may shift the balance towards a Th1 response with a proinflammatory action, increasing the risk of PD. Smithgall et al. [22] suggested that IL-33 primarily drives Th2 response but also promotes Th1 response under certain conditions; therefore, IL-33 can be a general amplifier of inflammation, with the outcome depending on the local cellular and extracellular context. Extracellular IL-33 promoted the production of cytokines and chemokines that increase neutrophil recruitment, NK cells, dendritic cell migration, and T lymphocyte polarization [23]. Furthermore, the IL-33 bound to ST2 has been shown to be associated with tissue destruction, transcription of factors related to T-cell activation, and proinflammatory cytokine secretion, resulting in the destruction of periodontal tissue [2]. In osteosarcoma patients, low levels of IL-33 were associated with protection when compared with controls [24]. Furthermore, in the model of rheumatoid arthritis that shares immunological mechanisms similar to PD, no effect was observed [25].

The IL-33 appears to be related to the bone remodeling process. Mun et al. [26] demonstrated that IL-33 can drive the differentiation of osteoclasts from CD14+ monocytes, activating monocyte phosphorylation signaling molecules and increasing the expression of osteoclast differentiating factors. However, in the presence of anti-ST2, these effects are suppressed. The expression of IL-33 can be induced by gingipain, fimbriae, and lipopeptide from *P. gingivalis* [8], and *P. gingivalis*-infected mice treated with IL-33 have shown greater alveolar bone loss when compared with isolation treatment, proposing a synergistic effect [27]. Added to this, under treatment with IL-33, the expression of RANKL by T and B lymphocytes was increased [27]. This highlights the role of the IL-33/ST2 axis in osteoclastogenesis. However, there is no consensus and studies have demonstrated that IL-33 may inhibit the formation of osteoclasts [28] and increase the production of osteoblasts [26], suggesting that IL-33 cannot be related to bone resorption [29]; rather, IL-33 may protect against bone resorption via RANKL suppression and OPG induction [30] and by enhancing GM-CSF, IL-4, IFN-*γ*, and TNF-*α* levels demonstrating protective effects in bone loss [31]. These conflicting data can make it difficult to understand the disease.

The *IL1RL1* rs3821024 G/G genotype, linked to reduced risk of PD, correlates with decreased sST2 levels compared to the C/C genotype due to the disruption of a miR202–3p binding site, destabilizing sST2 mRNA and decreasing sST2 expression [20]. Most of our patients have the genotype associated with higher production of sST2, and the binding of sST2/IL-33 prevents the production of Th2 cytokines, such as IL-4 and IL-5, but not Th1 cytokines [32], and it may favor an inflammatory profile. On the other hand, lower levels of sST2 in individuals with *IL1RL1* rs3821204 G/G genotype may favor the Th2 responses associated with protection in PD [33]. IL-33/sST2 has been related to the process of tissue homeostasis [34], acting in tissue repair with the alternative activation of macrophages (M2 subtype) and increased Treg ST2+ [35].

IL-33 serum levels had different concentrations between PD patients and controls, both nonsmokers. Patients exhibited a high production of IL-33, while in controls, the concentration was lower than the limit of detection, as reported by Saidi et al. [29]. This result reinforces the reliability of our samples, indicating that the control samples did not exhibit any previous inflammatory condition since IL-33 is produced constitutively and stored in the cell nucleus but under tissue necrosis, injury, damage, cellular stress conditions, or inflammatory signals, IL-33 production is increased and may be detectable in the serum [4, 36]. This may suggest that the higher concentration of IL-33 found in PD patients is due to the inflammatory process. Furthermore, several studies observed an increase in IL-33 concentration in gingival crevicular fluid, plasma, saliva [37], periodontal and gingival tissues [27], but not in the serum [38], suggesting that IL-33 is not related to systemic inflammation, but to local inflammation.

In this study, the risk of developing PD was observed in patients with genotypes associated with lower production of IL-33 (rs7025417) and higher production of sST2 (rs3821204). IL-33/ST2L plays an important role in Th2 responses; however, when IL-33 is bound to sST2, signaling is inhibited [22]. Therefore, our results may suggest that there is a switch from the Th2 to the Th1 pathway [32]. The Th1 pathway, as well as Th1-type cytokines, have been related to proinflammatory action, promoting tissue injury and bone resorption in PD [3].

To the best of our knowledge, this is the first study to analyze a potential association between the *IL1RL1* rs11685424 SNP and PD. Although our results indicate that *IL1RL1* rs11685424 is not related to the pathogenesis of PD, this SNP has been associated with respiratory conditions and heart diseases [21, 39]. Similarly, *IL33* rs1929992 did not show an association with PD, and a study conducted in a Saudi population supports this finding [40]. Nonetheless, these data are valuable for understanding the influence of IL-33 and its receptor on the development of the disease.

Among the positive points of the work, we highlight an efficient method of clinical selection of participants and the meticulous selection and noninclusion criteria that were used. Moreover, confounding factors were adjusted for multiple tests were applied. However, the study has its limitations. The low statistical power in nonsmokers and smokers, after stratification, can limit the identification of possible associations between them. In addition, it was not possible to measure sST2 levels, which could provide more conclusive results regarding the relationship between the IL-33/ST2 axis and the pathogenesis of PD. Furthermore, microbiological testing for *Aggregatibacter actinomycetemcomitans* was not performed, which could lead to confusion between patients with chronic and aggressive PD. Despite this, the classification was made by a specialized periodontist.

## 5. Conclusions

The *IL33* rs702541*7* polymorphism was associated with the risk of developing PD, and the *IL1RL1* rs3821204 SNP was associated with protection against PD in Brazilian individuals. No association was observed between *IL33* rs1929992 and *IL1RL1* rs11685424 polymorphisms and PD. Regarding serum levels of IL-33, our data support the hypothesis that IL-33 is increased in patients with PD; however, the production is independent of the extent and severity of the disease.

## Figures and Tables

**Table 1:** General characteristics of controls and patients with periodontitis.

	Controls	Patients	*p*-Value	OR (IC)
	*N* = 189	*N* = 186		
	*n* (%)	*n* (%)		
Age (years)				
Mean ± SD	45.17 ± 9.09	46.73 ± 9.09	>0.05	—
Gender				
Female	115 (60.8)	105 (56.5)	—	—
Male	74 (39.2)	81 (43.5)	>0.05	—
Smoking habits				
Nonsmokers	145 (76.7)	108 (58.1)	—	Ref.
Smokers	44 (23.3)	78 (41.9)	**0.0001**	**2.38 (1.52–3.71)**
Extension				
Generalized	—	46 (52.9)	—	—
Localized	—	41 (47.1)	—	—
Severity				
Slight	—	23 (26.5)	—	—
Moderate	—	35 (40.2)	—	—
Severe	—	29 (33.3)	—	—

*Note: N*, total number of controls or of patients with PD; *n*, number of individuals with the specific characteristic. Significant values are shown in bold, *p* < 0.05.

Abbreviations: CI, confidence interval; OR, odds ratio; SD, standard deviation.

**Table 2:** The *IL33* rs1929992, *IL33* rs7025417, *IL1RL1* rs11685424, and *IL1RL1* rs3821024 genotype and allele frequencies distribution in patients with PD and controls in general population and nonsmokers individuals adjusted by smoking habits, gender, and age.

	Nonsmokers *N* = 253					
Gene/polymorphism/inheritance model	Genotypes	Controls *n* (%)	Patients *n* (%)	*p*-Value	*Pc⁣* ^*∗*^	OR (IC)
*IL33 rs7025417 T>C*						
	T/T	99 (68.3)	68 (63)	—		Ref.
Codominant	T/C	45 (31)	31 (28.7)	—	—	1 (0.57–1.73)
	C/C	1 (7)	9 (8.3)	**0.006**	**0.015**	**12.85 (1.59**–**104.11)**
Recessive	T/T–T/C	144 (99.3)	99 (91.7)	—	—	Ref.
	C/C	1 (0.7)	9 (8.3)	**0.001**	**0.005**	**12.86 (1.60**–**103.49)**

	**General population *N* = 375**					

*IL33 rs1929992 T>C*						
	T/T	73 (38.6)	81 (43.5)	—	—	Ref.
Codominant	T/C	90 (47.6)	84 (45.2)	—	—	0.80 (0.51–1.25)
	C/C	26 (13.8)	21 (11.3)	0.47	—	0.70 (0.36–1.37)
Allele	T	236 (31.5)	246 (32.8)	—	—	Ref.
	C	142 (18.9)	126 (16.8)	0.32	—	1.17 (0.87–1.58)

*IL33 rs7025417 T>C*						
	T/T	130 (68.8)	116 (62.4)	—	—	Ref.
Codominant	T/C	55 (29.1)	57 (30.6)	—	—	1.18 (0.75–1.86)
	C/C	4 (2.1)	13 (7)	0.09	—	3.36 (1.04–10.89)
Allele	T	315 (42)	289 (38.5)	—	—	Ref.
	C	63 (8.4)	83 (11.1)	0.06	—	0.70 (0.48–1.01)

*IL1RL1-rs11685424 G>A*						
Codominant	G/G	49 (25.9)	55 (29.6)	—	—	Ref.
	G/A	96 (50.8)	87 (46.8)	—	—	0.78 (0.47–1.28)
	A/A	44 (23.3)	44 (23.7)	0.6	—	0.82 (0.46–1.48)
Allele	G	194 (25.9)	197 (26.3)	—	—	Ref.
	A	184 (24.5)	175 (23.3)	0.7	—	1.07 (0.80–1.42)

*IL1RL1 rs3821204 C>G*						
	T/T	122 (64.5)	124 (66.7)	—	—	Ref.
Codominant	T/C	56 (29.6)	59 (31.7)	—	—	1.01 (0.64–1.60)
	C/C	11 (5.8)	3 (1.6)	**0.026**	0.06	**0.19 (0.05**–**0.73)**
Recessive	T/T–T/C	178 (94.2)	183 (98.4)	—	—	Ref.
	C/C	11 (5.8)	3 (1.6)	**0.006**	**0.03**	**0.19 (0.05**–**0.72)**
Allele	C	300 (30)	307 (40.9)	—	—	Ref.
	G	78 (10.4)	65 (8.7)	0.31	—	1.23 (0.85–1.77)

*Note: N*, total number; *n*, number of individuals; *Pc⁣*^*∗*^, *p*-value corrected by false discovery rate (FDR) only for significant *p*-value; Ref., most frequently genotypes/allele (OR = 1). Significant values are shown in bold, *p* < 0.05.

Abbreviations: CI, confidence interval; OR, odds ratio.

**Table 3:** Cytokine levels in serum of patients with periodontitis, classified according to extension and severity, and controls.

	*n*	Mean (pg/mL)	SD
Controls	8	0	0
Patients	30	247.0	51.2
Slight	6	232.9	45.9
Moderate	10	260.5	70.1
Severe	14	243.3	37.3
Localized	15	254.2	47.5
Generalized	15	239.8	55.4

*Note: n*, number of individuals.

Abbreviation: SD, standard deviation.

## Data Availability

The datasets used and/or analyzed during the current study are available from the corresponding author upon reasonable request.

## Supporting Information

Supporting Information Tables S1–S3: Genotype frequencies distribution of *IL33* rs1929992 and rs7025417 and *IL1RL1* rs3821204 and rs11685424 genes in patients with PD and controls in general population, smokers and nonsmokers individuals considering the gender. Tables S4–S6: Genotype frequencies distribution of *IL33* rs1929992 and rs7025417 and *IL1RL1* rs3821204 and rs11685424 genes in patients with PD and controls in general population, smokers and nonsmokers individuals without association.
