# Supplementary material for: The Influence of Genetic Polymorphisms of IL33 and IL1RL1 Genes on the Immunopathogenesis of Periodontitis
Source: Int J Dent. 2025 May 2;2025:7599713. doi: 10.1155/ijod/7599713 (PMC12064316; doi:10.1155/ijod/7599713)
Supplement: Supporting Information — Tables S1–S3: Genotype frequencies distribution of IL33 rs1929992 and rs7025417 and IL1RL1 rs3821204 and rs11685424 genes in patients with PD and controls in general population, smokers and nonsmokers individuals considering the gender. Tables S4–S6: Genotype frequencies distribution of IL33 rs1929992 and rs7025417 and IL1RL1 rs3821204 and rs11685424 genes in patients with PD and controls in general population, smokers and nonsmokers individuals without association. [file 7599713.f1.docx]

Supplementary table 1: Genotype frequencies distribution of *IL33* rs1929992 and rs7025417 and *IL1RL1* rs3821204 and rs11685424 genes in patients with PD and controls in non-smokers considering the gender.

| Non-Smokers N= 253 |  |  |  |  |  |
| --- | --- | --- | --- | --- | --- |
| Gender | Genotype | Controls | Periodontitis | OR (CI) | *P* |
|  |  | n (%) | n (%) |  | value |
| *IL33 - rs1929992 T>C* |  |  |  |  |  |
| Women | T/T | 34 (36.5) | 30 (46.9) | Ref. |  |
|  | T/C | 46 (49.5) | 27 (42.1) | 0.66 (0.33-1.32) | 0,05 |
|  | C/C | 13 (14.0) | 7 (11.0) | 0.61 (0.21-1.73) |  |
| Men | T/T | 23 (44.2) | 21 (47.7) | Ref. |  |
|  | T/C | 20 (38.5) | 20 (45.5) | 1.12 (0.47-2.65) | 0,89 |
|  | C/C | 9 (17.3) | 3 (6.8) | 0.36 (0.09-1.53) |  |
| *IL33 - rs7025417 T>C* |  |  |  |  |  |
| Women | T/T | 63 (67.7) | 42 (65.6) | Ref. |  |
|  | T/C | 29 (31.2) | 18 (28.1) | 0.92 (0.45-1.87) | 0,91 |
|  | C/C | 1 (1.1) | 4 (6.3) | 6.28 (0.67-58.49) |  |
| Men | T/T | 36 (69.2) | 26 (59.10 | Ref. |  |
|  | T/C | 16 (30.80 | 13 (29.5) | 1.13 (0.46-2.75) | 0,41 |
|  | C/C | 0 (0.0) | 5 (11.4) |  |  |
| *IL33 - rs11685424 G>A* |  |  |  |  |  |
| Women | G/G | 26 (27.9) | 24 (37.5) | Ref. |  |
|  | G/A | 49 (52.7) | 23 (35.9) | 0.49 (0.23-1.03) | 0,27 |
|  | A/A | 18 (19.4) | 17 (26.9) | 1.00 (0.42-2.38) |  |
| Men | G/G | 10 (19.2) | 11 (25.0) | Ref. |  |
|  | G/A | 28 (53.9) | 25 (56.8) | 0.83 (0.30-2.28) | 0,66 |
|  | A/A | 14 (26.9) | 8 (18.2) | 0.49 (0.14-1.68) |  |
| *IL1RL1 - rs3821204 C>G* |  |  |  |  |  |
| Women | C/C | 65 (69.9) | 43 (67.2) | Ref. |  |
|  | C/G | 27 (29.0) | 20 (31.2) | 1.11 (0.55-2.23) | 0,85 |
|  | G/G | 1 (1.1) | 1 (1.6) | 1.61 (0.10-26.78) |  |
| Men | C/C | 32 (61.5) | 29 (65.9) | Ref. |  |
|  | C/G | 15 (28.9) | 15 (34.1) | 1.14 (0.47-2.75) | 0,81 |
|  | G/G | 5 (9.6) | 0 (0.0) |  |  |

OR: Odds Ratio, CI: Confidence Interval, N=total number, n: number of individuals, Ref.: Reference group.

Supplementary table 2: Genotype frequencies distribution of *IL33* rs1929992 and rs7025417 and *IL1RL1* rs3821204 and rs11685424 genes in patients with PD and controls in smokers considering the gender.

| Smokers N= 122 |  |  |  |  |  |
| --- | --- | --- | --- | --- | --- |
| Gender | Genotype | Controls | Periodontitis | OR (CI) | *P* |
|  |  | n (%) | n (%) |  | value |
| *IL33 - rs1929992 T>C* |  |  |  |  |  |
| Women | T/T | 9 (40.9) | 14 (34.1) | Ref. |  |
|  | T/C | 10 (45.5) | 22 (53.7) | 1.42 (0.45-4.44) | 0,79 |
|  | C/C | 3 (13.6) | 5 (12.2) | 0.98 (0.18-5.23) |  |
| Men | T/T | 7 (31.8) | 16 (43.2) | Ref. |  |
|  | T/C | 14 (63.3) | 15 (40.6) | 0.41 (0.13-1.34) | 0,55 |
|  | C/C | 1 (4.6) | 6 (16.2) | 2.57 (0.25-26.33) |  |
| *IL33 - rs7025417 T>C* |  |  |  |  |  |
| Women | T/T | 17 (77.3) | 23 (56.1) | Ref. |  |
|  | T/C | 4 (18.2) | 17 (41.5) | 3.02 (0.85-10.73) | 0,16 |
|  | C/C | 1 (4.5) | 1 (2.4) | 0.45 (0.02-8.46) |  |
| Men | T/T | 14 (63.6) | 25 (67.6) | Ref. |  |
|  | T/C | 6 (27.3) | 9 (24.3) | 1.10 (0.31-3.91) | 0,98 |
|  | C/C | 2 (9.1) | 3(8.1) | 0.70 (0.10-5.07) |  |
| *IL33 - rs11685424 G>A* |  |  |  |  |  |
| Women | G/G | 6 (27.3) | 9 (21.9) | Ref. |  |
|  | G/A | 9 (40.9) | 23 (56.2) | 1.73 (0.47-6.36) | 0,91 |
|  | A/A | 7 (31.8) | 9 (21.9) | 0.88 (0.21-3.75) |  |
| Men | G/G | 7 (31.8) | 11 (29.7) | Ref. |  |
|  | G/A | 10 (45.5) | 16 (43.2) | 1.02 (0.29-3.59) | 0,9 |
|  | A/A | 5 (22.7) | 10 (27.1) | 1.22 (0.28-5.23) |  |
| *IL1RL1 - rs3821204 C>G* |  |  |  |  |  |
| Women | C/C | 12 (54.6) | 23 (56.1) | Ref. |  |
|  | C/G | 8 (36.4) | 17 (41.5) | 1.15 (0.38-3.48) | 0,88 |
|  | G/G | 2 (1.0) | 1 (2.4) | 1.15 (0.38-3.48) |  |
| Men | C/C | 13 (59.1) | 29 (78.4) | Ref. |  |
|  | C/G | 6 (27.3) | 7 (18.9) | 0.38 (0.10-1.46) | 0,19 |
|  | G/G | 3 (13.6) | 1 (2.7) | 0.18 (0.02-1.91) |  |

OR: Odds Ratio, CI: Confidence Interval, N=total number, n: number of individuals, Ref.: Reference group.

Supplementary table 3: Genotype frequencies distribution of *IL33* rs1929992 and rs7025417 and *IL1RL1* rs3821204 and rs11685424 genes in patients with PD and controls in general population considering the gender.

|  |  | General population N= 375 | |  |  |
| --- | --- | --- | --- | --- | --- |
| Gender | Genotype | Controls | Periodontitis | OR (CI) | *P* |
|  |  | n (%) | n (%) |  | value |
| *IL33 - rs1929992 T>C* |  |  |  |  |  |
| Women | T/T | 43 (37.4) | 44 (41.9) | Ref. |  |
|  | T/C | 56 (48.7) | 49 (46.7) | 0.82 (0.46-1.46) | 0,81 |
|  | C/C | 16 (13.9) | 12 (11.4) | 0.70 (0.29-1.69) |  |
| Men | T/T | 30 (40.5) | 37 (45.7) | Ref. |  |
|  | T/C | 34 (45.9) | 35 (43.2) | 0.78 (0.39-1.56) | 0,58 |
|  | C/C | 10 (13.6) | 9 (11.1) | 0.70 (0.24-1.99) |  |
| *IL33 - rs7025417 T>C* |  |  |  |  |  |
| Women | T/T | 80 (69.6) | 65 (61.9) | Ref. |  |
|  | T/C | 33 (28.7) | 35 (33.3) | 1.26 (0.70-2.27) | 0,62 |
|  | C/C | 2 (1.7) | 5 (4.8) | 3.22 (0.58-17.87) |  |
| Men | T/T | 50 (67.6) | 51 (63) | Ref. |  |
|  | T/C | 22 (29.7) | 22 (27.1) | 1.07 (0.52-2.21) | 0,29 |
|  | C/C | 2 (2.7) | 8 (9.9) | 3.45 (0.68-17.51) |  |
| *IL33 - rs11685424 G>A* |  |  |  |  |  |
| Women | G/G | 32 (27.8) | 33 (31.4) | Ref. |  |
|  | G/A | 58 (50.4) | 46 (43.8) | 0.68 (0.36-1.29) | 0,66 |
|  | A/A | 25 (21.8) | 26 (24.8) | 0.91 (0.43-1.94) |  |
| Men | G/G | 17 (23) | 22 (27.2) | Ref. |  |
|  | G/A | 38 (51.3) | 41 (50.6) | 0.94 (0.43-2.08) | 0,66 |
|  | A/A | 19 (25.7) | 18 (22.2) | 0.72 (0.28-1.82) |  |
| *IL1RL1 - rs3821204 C>G* |  |  |  |  |  |
| Women | C/C | 77 (67) | 66 (62.9) | Ref. |  |
|  | C/G | 34 (29.5) | 37 (35.2) | 1.15 (0.64-2.07) | 0,67 |
|  | G/G | 4 (3.5) | 2 (1.9) | 0.42 (0.07-2.55) |  |
| Men | C/C | 45 (60.8) | 58 (71.6) | Ref. |  |
|  | C/G | 22 (29.7) | 22 (27.2) | 0.83 (0.40-1.73) | 0,62 |
|  | G/G | 7 (9.5) | 1 (1.2) | 0.09 (0.01-0.76) |  |

OR: Odds Ratio, CI: Confidence Interval, N=total number, n: number of individuals, Ref.: Reference group.

Supplementary table 4: Genotype frequencies distribution of *IL33* rs1929992 and rs7025417 and *IL1RL1* rs3821204 and rs11685424 genes in patients with PD and controls in non-smokers individuals without association.

|  |  | Non-smokers N= 253 |  |  |  |  |
| --- | --- | --- | --- | --- | --- | --- |
| Gene/Polymorphisms/ | Genotype/ | Controls | Periodontitis | *P* | *Pc* | OR (CI) |
| Inheritance model | Allele | n (%) | n (%) | value* |  |  |
| *IL33 - rs1929992 T>C* |  |  |  |  |  |  |
| Codominat | T/T | 57 (39.3) | 51 (47.2) |  |  | Ref. |
|  | T/C | 66 (45.5) | 47 (43.5) |  |  | 0.81 (0.47-1.38) |
|  | C/C | 22 (15.2) | 10 (9.3) | 0,27 | 0.34 | 0.51 (0.22-1.18) |
| Dominant | T/T | 57 (39.3) | 51 (47.2) |  |  | Ref. |
|  | T/C - C/C | 88 (60.7) | 57 (52.8) | 0,23 | 0.34 | 0.73 (0.44-1.22) |
| Recessivo | T/T - T/C | 123 (84.8) | 98 (90.7) |  |  | Ref. |
|  | C/C | 22 (15.2) | 10 (9.3) | 0,15 | 0.34 | 0.57 (0.26-1.26) |
| Overdominat | T/T - C/C | 79 (54.5) | 61 (56.5) |  |  | Ref. |
|  | T/C | 66 (45.5) | 47 (43.5) | 0,80 | 0.8 | 0.94 (0.57-1.55) |
| Log-additive |  |  |  | 0,12 | 0.34 | 0.74 (0.51-1.08) |
|  | T | 180 (62) | 149 (69) |  |  | Ref. |
|  | C | 110 (38) | 67 (31) | 0,12 |  | 1.36 (0.94-1.97) |
| HWE |  | 0,72 | 1,00 |  |  |  |
| *IL33 - rs7025417 T>C* |  |  |  |  |  |  |
| Codominat | T/T | 99 (68.3) | 68 (63) |  |  | Ref. |
|  | T/C | 45 (31) | 31 (28.7) |  |  | 1 (0,57 - 1,73) |
|  | C/C | 1 (7) | 9 (8.3) | **0.006** | **0.015** | **12,85 (1,59 - 104,11)** |
| Dominant | T/T | 99 (68.3) | 68 (63) |  |  | Ref. |
|  | T/C - C/C | 46 (31.7) | 40 (37) | 0,40 | 0.5 | 1.25 (0.74-2.12) |
| Recessive | T/T – T/C | 144 (99.3) | 99 (91.7) |  |  | Ref. |
|  | C/C | 1 (0.7) | 9 (8.3) | **0.001** | **0.005** | **12,86 (1,60 - 103,49)** |
| Overdominat | T/T - C/C | 100 (69) | 77 (71.3) |  |  | Ref. |
|  | T/C | 45 (31) | 31 (28.7) | 0,67 | 0.67 | 0.89 (0.51-1.54) |
| Log-additive |  |  |  | 0,08 | 0.13 | 1.49 (0.95-2.34) |
|  | T | 243 (84) | 167 (77) |  |  | Ref. |
|  | C | 47 (16) | 49 (23) | 0,08 |  | 0.66 (0.42-1.03) |
| HWE |  | 0,12 | 0,09 |  |  |  |
| *IL1RL1 - rs11685424 G>A* |  |  |  |  |  |  |
| Codominat | G/G | 36 (24.8) | 35 (32.4) |  |  | Ref. |
|  | G/A | 77 (53.1) | 48 (44.4) |  |  | 0.61 (0.34-1.10) |
|  | A/A | 32 (22.1) | 25 (23.1) | 0,26 | 0.43 | 0.76 (0.37-1.54) |
| Dominant | G/G | 36 (24.8) | 35 (32.4) |  |  | Ref. |
|  | G/A - A/A | 109 (75.2) | 73 (67.6) | 0,14 | 0.37 | 0.65 (0.37-1.14) |
| Recessivo | G/G - G/A | 113 (77.9) | 83 (76.8) |  |  | Ref. |
|  | A/A | 32 (22.1) | 25 (23.1) | 0,89 | 0.89 | 1.04 (0.57-1.90) |
| Overdominat | G/G - A/A | 68 (46.9) | 60 (55.6) |  |  | Ref. |
|  | G/A | 77 (53.1) | 48 (44.4) | 0,15 | 0.37 | 0.69 (0.42-1.14) |
| Log-additive |  |  |  | 0,39 | 0.48 | 0.85 (0.60-1.22) |
|  | G | 149 (51) | 118 (55) |  |  | Ref. |
|  | A | 141 (49) | 98 (45) | 0,52 |  | 1.14 (0.80-1.62) |
| HWE |  | 0,51 | 0,33 |  |  |  |
| *IL1RL1 - rs3821204 C>G* |  |  |  |  |  |  |
| Codominat | C/C | 97 (66.9) | 72 (66.7) |  |  | Ref. |
|  | C/G | 42 (29) | 35 (32.4) |  |  | 1.12 (0.65-1.93) |
|  | G/G | 6 (4.1) | 1 (0.9) | 0,16 | 0.40 | 0.18 (0.02-1.56) |
| Dominant | C/C | 97 (66.9) | 72 (66.7) |  |  | Ref. |
|  | C/G - G/G | 48 (33.1) | 36 (33.3) | 0,98 | 0.98 | 0.99 (0.58-1.69) |
| Recessivo | C/C - C/G | 139 (95.9) | 107 (99.1) |  |  | Ref. |
|  | G/G | 6 (4.1) | 1 (0.9) | 0,06 | 0.3 | 0.17 (0.02-1.50) |
| Overdominat | C/C - G/G | 103 (71) | 73 (67.6) |  |  | Ref. |
|  | C/G | 42 (29) | 35 (32.4) | 0,54 | 0.71 | 1.18 (0.69-2.03) |
| Log-additive |  |  |  | 0,57 | 0.71 | 0.87 (0.55-1.40) |
|  | C | 236 (81) | 179 (83) |  |  | Ref. |
|  | G | 54 (19) | 31 (17) | 0,31 |  | 1.32 (0.81-2.14) |
| HWE |  | 0,58 | 0,19 |  |  |  |

OR: Odds Ratio, CI: Confidence Interval, N=total number, n: number of individuals, HWE: Hardy Weinberg equilibrium, Ref.: Reference group, *Pc*= *P-value* corrected by False Discovery Rate (FDR).

Supplementary table 5: Genotype frequencies distribution of *IL33* rs1929992 and rs7025417 and *IL1RL1* rs3821204 and rs11685424 genes in patients with PD and controls in smokers individuals without association.

|  | Smokers N= | 122 |  |  |  |  |
| --- | --- | --- | --- | --- | --- | --- |
| Gene/Polymorphisms/ | Genotype/ | Controls | Periodontitis | *P* | *Pc* | OR (CI) |
| Inheritance model | Allele | n (%) | n (%) | value* |  |  |
| *IL33 - rs1929992 T>C* |  |  |  |  |  |  |
| Codominat | T/T | 16 (36.4) | 30 (38.5) |  |  | Ref. |
|  | T/C | 24 (54.5) | 37 (47.4) |  |  | 0.78 (0.35-1.75) |
|  | C/C | 4 (9.1) | 11 (14.1) | 0,62 | 0.88 | 1.38 (0.37-5.12) |
| Dominant | T/T | 16 (36.4) | 30 (38.5) |  |  | Ref. |
|  | T/C - C/C | 28 (63.6) | 48 (61.5) | 0,71 | 0.88 | 0.86 (0.40-1.88) |
| Recessivo | T/T - T/C | 40 (90.9) | 67 (85.9) |  |  | Ref. |
|  | C/C | 4 (9.1) | 11 (14.1) | 0,44 | 0.88 | 1.59 (0.47-5.41) |
| Overdominat | T/T - C/C | 20 (45.5) | 41 (52.6) |  |  | Ref. |
|  | T/C | 24 (54.5) | 37 (47.4) | 0,39 | 0.88 | 0.72 (0.34-1.53) |
| Log-additive |  |  |  | 0,92 | 0.92 | 1.03 (0.58-1.82) |
|  | T | 56 (64) | 97 (62) |  |  | Ref. |
|  | C | 32 (36) | 59 (38) | 0,93 |  | 0.94 (0.55-1.61) |
| HWE |  | 0,34 | 1,00 |  |  |  |
| *IL33 - rs7025417 T>C* |  |  |  |  |  |  |
| Codominat | T/T | 31 (70.5) | 48 (61.5) |  |  | Ref. |
|  | T/C | 10 (22.7) | 26 (33.3) |  |  | 1.87 (0.78-4.50) |
|  | C/C | 3 (6.8) | 4 (5.1) | 0,28 | 0.46 | 0.63 (0.12-3.32) |
| Dominant | T/T | 31 (70.5) | 48 (61.5) |  |  | Ref. |
|  | T/C - C/C | 13 (29.6) | 30 (38.5) | 0,28 | 0.46 | 1.55 (0.69-3.48) |
| Recessive | T/T - T/C | 41 (93.2) | 74 (94.9) |  |  | Ref. |
|  | C/C | 3 (6.8) | 4 (5.1) | 0,47 | 0.55 | 0.54 (0.11-2.80) |
| Overdominat | T/T - C/C | 34 (77.3) | 52 (66.7) |  |  | Ref. |
|  | T/C | 10 (22.7) | 26 (33.3) | 0,13 | 0.46 | 1.92 (0.80-4.59) |
| Log-additive |  |  |  | 0,55 | 0.55 | 1.22 (0.63-2.35) |
|  | T | 72 (82) | 122 (78) |  |  | Ref. |
|  | C | 16 (18) | 34 (22) | 0,62 |  | 0.80 (0.41-1.54) |
| HWE |  | 0,13 | 0,75 |  |  |  |
| *IL1RL1 - rs11685424 G>A* |  |  |  |  |  |  |
| Codominat | G/G | 13 (29.6) | 20 (25.6) |  |  | Ref. |
|  | G/A | 19 (43.2) | 39 (50) |  |  | 1.33 (0.54-3.30) |
|  | A/A | 12 (27.3) | 19 (24.4) | 0,77 | 0.96 | 1.02 (0.37-2.82) |
| Dominant | G/G | 13 (29.6) | 20 (25.6) |  |  | Ref. |
|  | G/A - A/A | 31 (70.5) | 58 (74.4) | 0,66 | 0.96 | 1.21 (0.52-2.80) |
| Recessivo | G/G - G/A | 32 (72.7) | 59 (75.6) |  |  | Ref. |
|  | A/A | 12 (27.3) | 19 (24.4) | 0,7 | 0.96 | 0.85 (0.36-1.99) |
| Overdominat | G/G - A/A | 25 (56.8) | 39 (50) |  |  | Ref. |
|  | G/A | 19 (43.2) | 39 (50) | 0,47 | 0.96 | 1.32 (0.62-2.82) |
| Log-additive |  |  |  | 0,97 | 0.97 | 1.01 (0.60-1.70) |
|  | G | 45 (51) | 79 (51) |  |  | Ref. |
|  | A | 43 (49) | 77 (49) | 0,99 |  | 0.98 (0.58-1.65) |
| HWE |  | 0,38 | 1,00 |  |  |  |
| *IL1RL1 - rs3821204 C>G* |  |  |  |  |  |  |
| Codominat | C/C | 25 (56.8) | 52 (66.7) |  |  | Ref. |
|  | C/G | 14 (31.8) | 24 (30.8) |  |  | 0.74 (0.32-1.73) |
|  | G/G | 5 (11.4) | 2 (2.6) | 0,11 | 0.18 | 0.18 (0.03-1.02) |
| Dominant | C/C | 25 (56.8) | 52 (66.7) |  |  | Ref. |
|  | C/G - G/G | 19 (43.2) | 26 (33.3) | 0,18 | 0.22 | 0.59 (0.26-1.29) |
| Recessivo | C/C - C/G | 39 (88.6) | 76 (97.4) |  |  | Ref. |
|  | G/G | 5 (11.4) | 2 (2.6) | 0,05 | 0.17 | 0.20 (0.04-1.10) |
| Overdominat | C/C - G/G | 30 (68.2) | 54 (69.2) |  |  | Ref. |
|  | C/G | 14 (31.8) | 24 (30.8) | 0,72 | 0.72 | 0.86 (0.37-1.98) |
| Log-additive |  |  |  | 0,07 | 0.17 | 0.56 (0.29-1.05) |
|  | C | 64 (73) | 128 (82) |  |  | Ref. |
|  | G | 24 (27) | 28 (18) | 0,12 |  | 1.71( 0.92-3.19) |
| HWE |  | 0,25 | 1,00 |  |  |  |

OR: Odds Ratio, CI: Confidence Interval, N=total number, n: number of individuals, HWE: Hardy Weinberg equilibrium, Ref.: Reference group, *Pc*= *P-value* corrected by False Discovery Rate (FDR).

Supplementary table 6: Genotype frequencies distribution of *IL33* rs1929992 and rs7025417 and *IL1RL1* rs3821204 and rs11685424 genes in patients with PD and controls in general population individuals without association.

|  |  | General population N= 375 |  |  |  |  |
| --- | --- | --- | --- | --- | --- | --- |
| Gene/Polymorphisms/ | Genotype/ | Controls | Periodontitis | *P* | *Pc* | OR (CI) |
| Inheritance model | Allele | n (%) | n (%) | value* |  |  |
| *IL33 - rs1929992 T>C* |  |  |  |  |  |  |
| Codominat | T/T | 73 (38.6) | 81 (43.5) |  |  | Ref. |
|  | T/C | 90 (47.6) | 84 (45.2) |  |  | 0.80 (0.51-1.25) |
|  | C/C | 26 (13.8) | 21 (11.3) | 0,47 | 0.51 | 0.70 (0.36-1.37) |
| Dominant | T/T | 73 (38.6) | 81 (43.5) |  |  | Ref. |
|  | T/C - C/C | 116 (61.4) | 105 (56.5) | 0,24 | 0.51 | 0.78 (0.51-1.19) |
| Recessivo | T/T - T/C | 163 (86.2) | 165 (88.7) |  |  | Ref. |
|  | C/C | 26 (13.8) | 21 (11.3) | 0,46 | 0.51 | 0.79 (0.42-1.48) |
| Overdominat | T/T - C/C | 99 (52.4) | 102 (54.8) |  |  | Ref. |
|  | T/C | 90 (47.6) | 84 (45.2) | 0,51 | 0.51 | 0.87 (0.57-1.32) |
| Log-additive |  |  |  | 0,22 | 0.51 | 0.83 (0.61-1.13) |
|  | T | 236 (62) | 246 (66) |  |  | Ref. |
|  | C | 142 (38) | 126 (34) | 0,33 |  | 1.17 (0.87-1.58) |
| HWE |  | 0,88 | 1,00 |  |  |  |
| *IL33 - rs7025417 T>C* |  |  |  |  |  |  |
| Codominat | T/T | 130 (68.8) | 116 (62.4) |  |  | Ref. |
|  | T/C | 55 (29.1) | 57 (30.6) |  |  | 1.18 (0.75-1.86) |
|  | C/C | 4 (2.1) | 13 (7) | 0,09 | 0.15 | 3.36 (1.04-10.89) |
| Dominant | T/T | 130 (68.8) | 116 (62.4) |  |  | Ref. |
|  | T/C - C/C | 59 (31.2) | 70 (37.6) | 0,2 | 0.25 | 1.33 (0.86-2.06) |
| Recessive | T/T - T/C | 185 (97.9) | 173 (93) |  |  | Ref. |
|  | C/C | 4 (2.1) | 13 (7) | 0,04 | 0.15 | 3.19 (0.99-10.25) |
| Overdominat | T/T - C/C | 134 (70.9) | 129 (69.3) |  |  | Ref. |
|  | T/C | 55 (29.1) | 57 (30.6) | 0,68 | 0.68 | 1.10 (0.70-1.73) |
| Log-additive |  |  |  | 0,07 | 0.15 | 1.40 (0.97-2.03) |
|  | T | 315 (83) | 289 (78) |  |  | Ref. |
|  | C | 63 (17) | 83 (22) | 0,06 |  | 0.70 (0.48-1.00) |
| HWE |  | 0,79 | 0,14 |  |  |  |
| *IL1RL1 - rs11685424 G>A* |  |  |  |  |  |  |
| Codominat | G/G | 49 (25.9) | 55 (29.6) |  |  | Ref. |
|  | G/A | 96 (50.8) | 87 (46.8) |  |  | 0.78 (0.47-1.28) |
|  | A/A | 44 (23.3) | 44 (23.7) | 0,6 | 0.75 | 0.82 (0.46-1.48) |
| Dominant | G/G | 49 (25.9) | 55 (29.6) |  |  | Ref. |
|  | G/A - A/A | 140 (74.1) | 131 (70.4) | 0,33 | 0.75 | 0.79 (0.50-1.26) |
| Recessivo | G/G - G/A | 145 (76.7) | 142 (76.3) |  |  | Ref. |
|  | A/A | 44 (23.3) | 44 (23.7) | 0,9 | 0.9 | 0.97 (0.59-1.58) |
| Overdominat | G/G - A/A | 93 (49.2) | 99 (53.2) |  |  | Ref. |
|  | G/A | 96 (50.8) | 87 (46.8) | 0,44 | 0.75 | 0.85 (0.56-1.29) |
| Log-additive |  |  |  | 0,49 | 0.75 | 0.90 (0.67-1.21) |
|  | G | 194 (51) | 197 (53) |  |  | Ref. |
|  | A | 184 (49) | 175 (47) | 0,71 |  | 1.07 (0.80-1.42) |
| HWE |  | 0,88 | 0,46 |  |  |  |
| *IL1RL1 - rs3821204 C>G* |  |  |  |  |  |  |
| Codominat | T/T | 122 (64.5) | 124 (66.7) |  |  | Ref. |
|  | T/C | 56 (29.6) | 59 (31.7) |  |  | 1,01 (0,64 - 1,60) |
|  | C/C | 11 (5.8) | 3 (1.6) | **0.026** | 0.06 | **0,19 (0,05 - 0,73)** |
| Dominant | C/C | 122 (64.5) | 124 (66.7) |  |  | Ref. |
|  | C/G - G/G | 67 (35.5) | 62 (33.3) | 0,51 | 0.63 | 0.86 (0.56-1.34) |
| Recessivo | T/T – T/C | 178 (94.2) | 183 (98.4) |  |  | Ref. |
|  | C/C | 11 (5.8) | 3 (1.6) | **0.006** | **0.03** | **0,19 (0,05 - 0,72)** |
| Overdominat | C/C - G/G | 133 (70.4) | 127 (68.3) |  |  | Ref. |
|  | C/G | 56 (29.6) | 59 (31.7) | 0,69 | 0.69 | 1.10 (0.70-1.72) |
| Log-additive |  |  |  | 0,14 | 0.23 | 0.75 (0.52-1.10) |
|  | C | 300 (79) | 307 (83) |  |  | Ref. |
|  | G | 78 (21) | 65 (17) | 0,31 |  | 1.22 (0.85-1.77) |
| HWE |  | 0,19 | 0,21 |  |  |  |

OR: Odds Ratio, CI: Confidence Interval, N=total number, n: number of individuals, HWE: Hardy Weinberg equilibrium, Ref.: Reference group, *Pc*= *P-value* corrected by False Discovery Rate (FDR).
